# The Elevation of LC-ESI-Q-TOF-MS Response in the Analysis of Isoquinoline Alkaloids from Some Papaveraceae and Berberidaceae Representatives

**DOI:** 10.1155/2017/8384107

**Published:** 2017-12-25

**Authors:** Wirginia Kukula-Koch

**Affiliations:** Chair and Department of Pharmacognosy with Medicinal Plants Unit, 1 Chodzki St., 20-093 Lublin, Poland

## Abstract

Twenty-five methanol extracts obtained from various representatives of Papaveraceae and Berberidaceae botanical families (genera: *Papaver*, *Argemone*, *Eschscholzia*, *Chelidonium*, *Glaucium*, and *Berberis*) were screened for their alkaloid content in an optimized method suitable for the LC-ESI-Q-TOF-MS analysis. Twelve pharmacologically important isoquinoline alkaloids from four groups, aporphines, benzylisoquinolines, protoberberines, and benzophenanthridines, present in these traditionally used plant species were quantitatively determined in each studied sample, providing their alkaloid profile. A Zorbax Stable Bond RP-18 column and a mobile phase composed of 0.1% formic acid and 0.1% formic acid in acetonitrile (v/v) were used at the flow rate of 0.2 mL/min. A profound study on the optimization of MS response to four groups of isoquinoline alkaloids (validation of capillary voltage, gas flows, nebulizer pressure, skimmer, and fragmentor voltages), repeatability of results, and stability and linearity of measurements were described, showing, among others, 3000 V of capillary voltage, 350°C of gas temperature, 12 L/min of gas flows, nebulizer pressure of 35 psig, 65 V for skimmer voltage, and 30 V for collision energy as the most advantageous operation parameters.

## 1. Introduction

Isoquinoline alkaloids (ISQAs)—nitrogen containing secondary metabolites derived from the two amino acids phenylalanine and tyrosine—are the largest group of natural products. They are known from their prominent pharmacological activities and vast applications in modern pharmacotherapeutical strategies as single compounds or in pharmaceutical preparations from the whole plants [1]. Among ISQAs, morphine (an opioid analgesic drug) [2], berberine (a digestive system stimulant and antiprotozoal, antinociceptive, and neuroprotective agent) [3], boldine (a bile production stimulant), codeine (analgesic and antitussive remedy) [4], galanthamine (a competitive reversible acetylcholinesterase inhibitor used in mild and moderate dementia) [5], or papaverine (antispasmotic agent) should be listed as commonly used medicines [2].

Even if ISQAs comprise a variety of chemical structures with different levels of oxygenation, additional rings, and substituents, their basic skeleton always contains an isoquinoline moiety or a tetrahydroisoquinoline ring [4].

ISQAs may be divided into eight subgroups, four of which were evaluated in the presented study: protoberberines (which account for 25% of all ISQAs), aporphines, benzophenanthridines, and benzylisoquinolines [4, 6]. Described alkaloids are widespread in nature, although their highest content can be found in plants, in various representatives of Berberidaceae, Papaveraceae, Ranunculaceae, Rutaceae, Fumariaceae, or Menispermaceae [1, 6]. Due to significant pharmacological importance of these alkaloids, a thorough validation of mass spectrometry conditions in their analysis, directly in plant extracts, is presented in this manuscript. It is worth to note that still some of them are obtained from natural sources, which encourages the authors to discuss on their determination in the extracts and later on the purification by various separation techniques [4].

Based on the well-established applications in traditional medicine and a variety of ISQAs confirmed in their extracts, several Berberidaceae and Papaveraceae representatives have been selected for the purpose of this study. The importance and use of plant species belonging to the genus *Papaver* has a very long history dating back some 3,000 years, when it was already administered in the form of opium (containing a complex of alkaloids) as a pain-relieving remedy. Besides, various species of poppies have been used in the treatment of upper respiratory tract diseases, cough, and hoarseness and to facilitate the expulsion of mucus [7]. Barberry species are commonly administered bile production inducers. These widespread shrubs also exhibit antipyretic, anti-inflammatory, antidiarrheal, and hepatoprotective properties [8] and are present in a variety of traditional formulations and in homeopathy.

Even if these plants have been used for ages in traditional pharmacotherapeutical strategies, still multiple studies on their novel pharmacological applications are conducted, revealing new possible applications of their constituents, such as neuroprotective or anticancer activities [5, 8].

In the light of these findings, there is a strong need for the development of new, more accurate, specific, and precise analytical methods for their characterization. Electrospray (ESI) mass spectrometry is a sensitive analytical tool, which enables the determination of single compounds in a complex matrix. It was found to deliver high-accuracy data, which provided a successful identification of isoquinolines of different kind [9–11]. The aim of this manuscript is to carefully design the operation parameters of a high-resolution mass spectrometer, LC-ESI-Q-TOF-MS, such as fragmentation energies, gas flows and temperatures, skimmer voltage, and nebulizer pressure, to deliver high-accuracy qualitative and quantitative data for the determination of ISQAs in plant materials. The analyses will be performed on four model alkaloids—the representatives of different subgroups of ISQAs to indicate their analytical behaviour. Because of a high popularity and a marked sensitivity of this particular type of mass spectrometer, the optimization results may be applied in various plant species from the abovementioned botanical families.

Additionally, the determination of some selected alkaloids under the optimized analytical conditions will be performed on 25 extracts obtained from poppy and barberry species to create their chemotaxonomical fingerprint and fish the most valuable ones—the most rich in alkaloids.

## 2. Materials and Methods

### 2.1. Reagents

Acetonitrile, water, and formic acid of spectroscopic purity were obtained from J. T. Baker (Center Valley, PA, USA), whereas the extraction solvents were obtained from Avantor Performance Materials (Gliwice, Poland). 0.45 µm nylon syringe filters (Merck, Darmstadt, Germany) were used for the filtration of the obtained extracts at given concentrations. Reference compounds were purchased from ChromaDex Inc. (Irvine, CA, USA).

### 2.2. Plant Material

Various species of Berberidaceae and Papaveraceae (Table 1) were cut (aerial parts after the flowering, underground parts in the late September before the frost), dried at 40°C after collection, finely powdered, and frozen prior to the study. A sample of each plant was stored by the author under voucher specimen numbers WKK2016M001–WKK2016M025.

All poppies were cultivated in the garden of the Department of Pharmacognosy, Medical University of Lublin. The seeds were obtained from botanical gardens around the world, which the Department of Pharmacognosy collaborates with, for many years now. The plants were additionally authenticated by the author. The root of *Berberis vulgaris* was purchased in a local herbal shop in Lublin, Poland. The overground parts of *Berberis siberica* were obtained from Mongolia (from the area of Ulaanbaatar in September 2015) and were authenticated by Dr. Otgonbataar Urjin. *Berberis cretica* came from Crete island in Greece (the surroundings of Iraklion in September 2015) and was authenticated by the author. The list of species used in the study is given in Table 1.

### 2.3. Extraction

1 g of powdered plant material was extracted by accelerated solvent extraction (ASE) in the apparatus produced by Dionex (ASE 100, Sunnyvale, CA, US) locked in a 10 mL stainless steel vessel, under the following conditions: extractant: methanol; temperature: 80°C; static time: 5 min; number of cycles: 1; flush volume: 50%; purge time: 50 s. Pressure was maintained at ca 110 bar. The extraction for each plant species was performed 3 times. The obtained extracts were diluted to the volume of 20 mL, and 1.5 mL of each was transferred to an HPLC vial through a nylon syringe filter and subjected to LC-MS analysis. The remaining samples were dried under vacuum in 50°C until dryness.

### 2.4. Qualitative and Quantitative High-Resolution Mass Spectrometry Analysis of the Extracts

An LC-ESI-Q-TOF-MS apparatus produced by Agilent Technologies (Santa Clara, CA, US) was applied in the study. The instrument was composed of an HPLC chromatograph (1260 Series) containing a degasser (G1322A), a binary pump (G1312C), an autosampler (G1329B), a column oven (G1316A), a PDA detector (G1315D), and a mass spectrometer (G6530B) with a quadrupole and a time-of-flight analyzers.

To obtain high-resolution spectra of alkaloids, positive ionisation mode was applied. A gradient of acetonitrile (*B*) and water (*A*), both with an addition of formic acid, was carefully adjusted to provide sufficient separation of the extract on a chromatographic column (Zorbax Stable Bond RP-18 Column: 150 mm × 2.1 mm, dp = 3.5 µm): 0 min–1% *B* in *A*, 10 min–40% *B* in *A*, 12 min–40% *B* in *A*, 17 min–95% *B* in *A*, 20 min–1% *B* in *A*, stop time: 30 min. 0.2 mL/min flow rate, 5 min post time, 10 µL injection volume, and the *m/z* range from 40 to 1000 were set in the method details. The analysis was performed at 25°C [10] on a freshly calibrated apparatus. Two most intensive signals were simultaneously fragmented in the MS/MS analysis. After the collection of 1 spectrum, they were excluded for 0.3 min from the analysis to collect more fragmentation data from other less intense signals. A calibration mixture was dosed simultaneously during the analysis as an internal standard to sustain high accuracy of the measurements.

To provide the quantitative analysis of alkaloids, four reference compounds—representatives of each class of studied alkaloids—were evaluated: berberine (a protoberberine alkaloid), berbamine (a benzylisoquinoline alkaloid), sanguinarine (a benzophenanthridine type), and magnoflorine (an aporphine alkaloid). All of them were obtained from Chromadex (Irvine, CA, US) at a purity higher than 95%. The calibration curve equations and *R*^2^ values were calculated for each reference compound from 6 separate injections of different concentrations at the same injection volume.

The MS parameters' optimization study was performed on the most diverse extract, methanol extract from *Berberis siberica*, based on the three separate injections for each changed parameter. Majority of the MS parameter settings were validated: capillary voltage varied from 3000 to 4000 V, fragmentor energy from 75 to 175, gas temperatures from 300°C to 400°C, drying gas flows from 10 to 12 L/min, nebulizer from 25 to 40 psig, skimmer from 40 to 70 V, and collision energies from 10 to 80 V (Table 2).

The following factors were included in the validation of the best evaluated method: the limit of detection (LOD) expressed as signal-to-noise (S/N) times 3 (measured in the vicinity of the peak of interest), the limit of quantification (LOQ) calculated as S/N times 10 [12], linearity (linear range determination), and precision deviation (interday and intraday variations) determined for *n* = 5 peak area measurements recorded once on the same day and another time at the very end of the sequence after 48 hours from the first injection (Table 3).

MassHunter B.07.00 software enabled both the conduction of analyses and the management of the obtained spectral data.

## 3. Discussion

### 3.1. The Validation of LC-ESI-Q-TOF-MS Operation Parameters

The aim of the study was to show the behaviour of four model representatives of different subgroups of isoquinoline alkaloids: BBR, which belongs to protoberberines; SNG, a benzophenanthridine alkaloid; MGN, an aporphine alkaloid; and BBM, a benzylisoquinoline alkaloid, in various conditions of mass spectrometric analysis. Unlike the commonly described optimization parameters, like stability, repeatability, or precision deviation assessment for the performed tests, the evaluation of general mass spectrometer settings is more rare in the scientific literature. Often, universal MS settings characteristic for each analytical laboratory are applied also for the determination of various secondary metabolites, including alkaloids, giving less accurate MS measurements. The author's intention was to present the influence of different operating parameters like temperature, pressure, and voltage on the abundance of alkaloids of interest in the analyzed extracts. Table 2 shows the percentage differences between the peak areas of 4 alkaloids determined in the Siberian barberry herb methanolic extract, in different spectrometric conditions applied. For this purpose, the highest peak area of each group was assigned as 100% intensity, and the others were calculated in relation to the former. The deviations in the peak area measurements were observed on a spectrum of this complex matrix of *B. siberica* extract to provide standard analysis conditions, which differ greatly from those of single reference compounds' injections. As it is presented in Table 2, the majority of selected parameters determine the final response of the instrument dependent on the sample ionisation efficiency.

Except from capillary voltage, all determined parameters were found to affect the injected extract in a similar way. The study revealed that berbamine—a representative of benzylisoquinoline alkaloids—delivered a better abundance in the higher capillary voltage. This behaviour might be explained by its dimeric structure, higher molecular weight, and stronger energy needed to transform the molecule into an ion. The other tested alkaloids showed an opposite reaction. To well center the applied conditions, the energy of 3500 V was selected as the most promising and supporting the highest abundances of all groups of compounds. In the case of three remaining compounds (BBR, SNG, and MGN), the decrease of capillary voltage below 3000 V did not bring better results.

Also, other MS instrument parameters influenced the quantification of the sample markedly. According to the results presented in Table 2, the fragmentor voltage was important for the generation of ions from the injected matrix. The lower its voltage, the smaller the quantity of ions detected by the analyzer. On the other hand, high voltage, such as 175 V at the instrument entrance, even if providing the highest peak areas, led to a faster fragmentation of alkaloids already in source, resulting in more crowded MS spectra. Considering the above, 150 V energy was used in the following analyses.

Surprisingly, the parameters of the carrier gases did not play any major role in the determination of these alkaloids. The temperature of gas and sheath gas, tested in the range of 300–375°, similarly to their flows (from 10 to 12 L/min) induced only minor, insignificant deviations.

On the other hand, the nebulizer pressure and the skimmer voltage were found to influence the analysis greatly. As much as an average 35% drop in the peak areas of all alkaloids was calculated for the pressure of 25 psig in relation to 35 psig. Aporphines were found to be the most sensitive for the change of this parameter. The application of 35 and 40 psig resulted in the highest response of the apparatus. The peak area of berbamine was the most resistant to the applied energies.

Skimmer voltage of 60 and 65 V was found to be the most beneficial, contrary to 40 V, which occurred to be a boarder setting, above which, a significant rise in the operation sensitivity was observed.

The last parameter to be optimized was the CID energy corresponding to the formation of the MS/MS spectra. A range of collision energies from 10 to 80 V was tested in the search for good fragmentation of these alkaloids. The results showed noticeable differences between the spectra depending on the CID energy applied. A sufficient fragmentation pattern for these alkaloids was obtained already in 30 V, still providing the presence of a molecular peak.

Lower energy values showed very weak fragmentation, when the highest used, crashed the structure into very small fragments making the identification troublesome. The list of obtained fragments for all tested energies is presented in Table 4.

Considering the above observations, the following analytical method including the best conditions for the alkaloid determination was composed: capillary voltage 3500 V, fragmentor energy from v 150, gas temperatures from 350°C, drying gas flows from 12 L/min, nebulizer from 35 psig, skimmer from 65 V, and collision energies CID from 20 to 30 V. Even if all described conditions are close to one another, the validated method parameters would certainly provide better results.

Some scientific papers show the determination of isoquinoline alkaloids in the samples of a different origin, like human plasma, molluscs, or different TCM preparations containing a variety of plant extracts. In a study of Yuan et al. [13], the determination of alkaloids was performed in both positive and negative ionisation modes in the capillary voltage of 3000 V, drying gas temperature of 325°C, and nebulizing gas pressure of 350 set for the study of yanhusuo herb. Shi et al. [14] applied 3000 V of capillary voltage, collision energy of 35 V, and the fragmentation energy of 6 V in a study on the rat plasma constituents. Also Zhou et al. [15] used an LC-MS apparatus for the determination of isoquinolines. In their research on the *Lotus nelumbo* compounds, the scientists applied 3000 V on a capillary and the temperature at 300°C to identify the constituents characterized by an anti-inflammatory action. In the light of the above discussed optimization, the previously published parameters, even if correct, seem not to use the maximum accuracy of the spectrometers.

### 3.2. Method Validation

The LOD and LOQ values were calculated for each alkaloid by a series of sample dilutions injected at the volume of 10 µL under the optimized parameters. The former was calculated as S/N 3 : 1, the latter as S/N of 10 : 1 according to Saadati et al. [12].

All measured values of LODs were far below the linearity ranges for matching alkaloids as indicated in Table 3 and varied between 18 and 47 ng/mL. The measured LOQs stayed within the range of 61–157 ng/mL. All drawn calibration curves of reference compounds were found to have a good linearity with correlation coefficient values higher than 0.997. In the optimized method, the response of apparatus to small concentrations of alkaloids was high so that the linearity range is within 0.1–250 µg/mL, similarly to those determined on other apparatus [10]. The deviation of results obtained in the interday and intraday precision measurements of all alkaloids were below 5%, which confirms high stability of the method.

### 3.3. Determination of Alkaloids in the Selected Extracts of Papaveraceae and Berberidaceae Representatives

**Figure 1:**
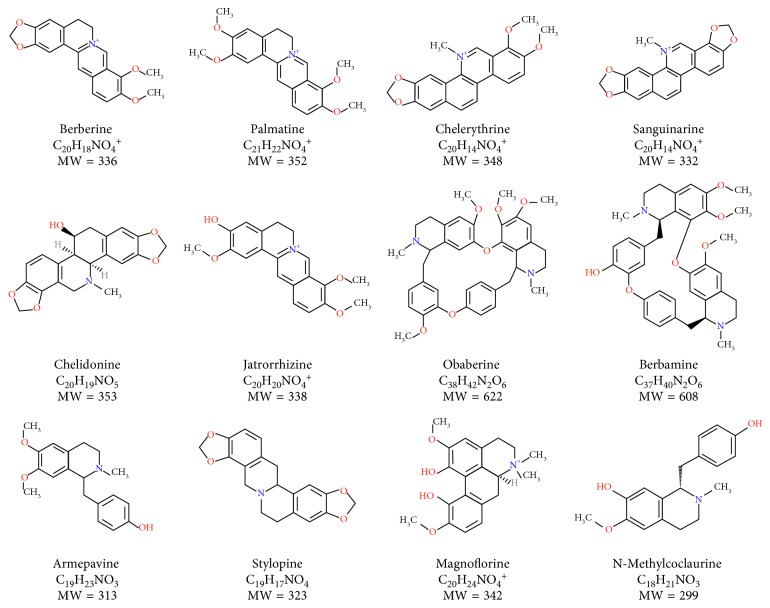
The chemical structures of the quantified isoquinoline alkaloids with their molecular weights and chemical formulas.

**Figure 2:**
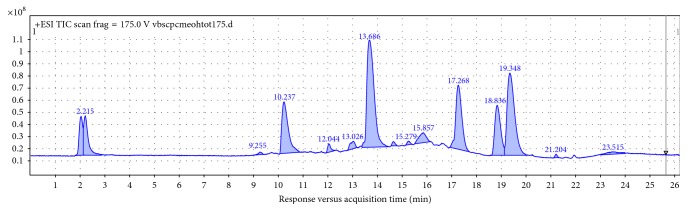
TIC of a sample extract: *Berberis cretica* methanol root extract in the optimized LC-MS method with BBR at 19.3 min, PLM at 18.8 min, JTR at 17.2 min, OBB at 16.6 min, STP at 15.8 min, BBM at 15.2 min, ARM at 14.6 min, and MGN at 13.7 min.

25 obtained extracts from selected Papaveraceae (genera: *Papaver*, *Eschscholzia*, *Chelidonium*, *Argemone*, and *Glaucium*) and Berberidaceae species (*Berberis* spp.) were evaluated for their qualitative and quantitative composition in the optimized method. According to the scientific literature, the following alkaloids from 4 groups of ISQAs were expected to occur in the analyzed samples: BBR, JTR, STP, SNG, CHE, ARM, BBM, OBB, and CHD (Figures 1 and 2) [16, 17]. All of them were identified, using the validated method, as separate peaks, in accordance with the scientific literature, their fragmentation patterns, Metlin database, and the behaviour of reference compounds. All compounds were quantified using the formerly described calibration curves (Table 3) of four model alkaloids. The data are presented in Table 3.

The listed compounds have been selected due to their significant pharmacological activities and a growing interest in their purification and activity assessment, to introduce the studied plant species as potential sources for their purification. Berberine, as the most widespread compound, has been already thoroughly studied. However, the remaining compounds still demand more attention and precise phytochemical profile assessment.

BBR was detected in all plant species from Papaveraceae and Berberidaceae and may be treated as a chemotaxonomic marker of these families. As described by Jensen and Kadereit [18], this alkaloid is present in all Ranunculiflorae—in Berberidaceae, Fumariaceae, Menispermaceae, Papaveraceae, and Ranunculalceae representatives. The extracts from barberry species did not reveal any presence of SNG, CHE, or CHD contrary to the poppies.

BBM and OBB were more abundant in barberry species, especially in their underground parts, whereas aporphines were spread more equally between the representatives of two families. PLT accompanied BBR in majority of extracts, whereas STP was present more often in the studied barberry species. Root samples contained higher concentrations of the produced alkaloids; however, still a higher diversity of compounds was noted in the green parts of the plants. *Berberis* root extracts belonged to the least diverse ones, but the quantities of identified alkaloids in their samples were high. *Berberis cretica* contained the highest quantity of MGN (∼7%) (Figure 2), and *Berberis vulgaris* contained the highest quantity of BBR (∼5.7%). *Papaver nudicaule* was the most rich in BBR among the poppies, *Eschscholzia californica* contained the highest amount (4.6%) of JTR and STP (3.7%) in the herb, and *Papaver pseudo-orientale* was characterized by a high content of PLT (5.7%)—the highest among all tested extracts. Poppies were found to contain two out of three quantified benzophenanthridine alkaloids: SNG (*P. caucasicum* with the highest content) and CHE (*Argemone grandiflora* herb). The least abundant compound among all tested extracts was N-methylcoclaurine—an aporphine found in *P. pillosum* in the highest concentration of 1.5% (Table 5).

## 4. Conclusions

The evaluated LC-ESI-Q-TOF-MS methodology led to a successful determination of 12 pharmacologically important isoquinoline alkaloids in 25 methanolic extracts from Papaveraceae and Berberidaceae representatives, with high accuracy, stability, and repeatability of results. The quantitative analysis of selected alkaloids in many plant extracts indicated new potential sources for the isolation of these pharmacologically valuable molecules.

## Figures and Tables

**Table 1:** A list of studied samples.

Extract number	Species name	Plant organ	Year of coll.
1	*Chelidonium majus*	*Herba*	2013
2	*Argemone ochroleuca*	*Herba*	2013
3	*Papaver pseudo-orientale*	*Folium*	2015
4	*Papaver caucasicum*	*Herba*	2013
5	*Papaver nudicaule*	*Herba*	2013
6	*Papaver crocetum*	*Herba*	2014
7	*Papaver bracteatum “Arya”*	*Folium*	2015
8	*Argemone mexicana*	*Herba*	2013
9	*Papaver pillosum*	*Folium*	2012
10	*Papaver argemone*	*Herba*	2011
11	*Papaver rhoeas*	*Herba*	2012
12	*Eschscholzia californica*	*Herba*	2012
13	*Papaver orientale*	*Folium*	2012
14	*Papaver lapponicum*	*Herba*	2012
15	*Glaucium flavum*	*Herba*	2012
16	*Berberis siberica*	*Herba*	2015
17	*Argemone grandiflora*	*Herba*	2008
18	*Berberis cretica*	*Herba*	2015
19	*Papaver pseudo-orientale*	*Radix*	2015
20	*Eschscholzia californica*	*Radix*	2014
21	*Argemone mexicana*	*Radix*	2011
22	*Chelidonium majus*	*Radix*	2009
23	*Argemone grandiflora*	*Radix*	2011
24	*Berberis cretica*	*Radix*	2015
25	*Berberis vulgaris*	*Radix*	2015

**Table 2:** The influence of spectrometric parameters on the determination of the studied four representatives of isoquinoline alkaloids, expressed in the peak area of the compounds in relation to the highest one in each group (in percent). The most optimum settings for all groups of alkaloids are in italics.

Instrument settings		% BBR	% BBM	% MGN	% SNG
Capillary voltage (V)	A3000	100.00	54.88	100.00	100.00
	A3250	98.77	66.97	92.28	96.25
	*A3500*	*97.19*	*96.48*	*90.23*	*95.17*
	A3750	82.83	100.00	81.28	84.77
	A4000	82.80	99.93	77.76	81.96

Fragmentor voltage (V)	75	52.21	62.47	55.49	58.39
	100	70.36	64.59	76.28	66.37
	125	85.44	69.81	95.08	87.35
	*150*	*98.46*	*95.72*	*99.95*	*99.02*
	*175*	*100.00*	*100.00*	*100.00*	*100.00*

Gas temperatures (°C)	*300*	*99.96*	*99.94*	*100.00*	*98.91*
	*325*	*100.00*	*100.00*	*99.90*	*100.00*
	*350*	*99.37*	*99.62*	*99.92*	*99.36*
	350 and 375 (sheath gas)	97.77	80.09	99.87	95.44

Drying gas and sheath gas flows (L/min)	10 and 10	95.65	92.98	95.09	96.12
	11 and 11	97.55	97.39	99.77	98.30
	*12 and 12*	*100.00*	*100.00*	*100.00*	*100.00*

Nebulizer pressure (psig)	25	63.44	77.86	52.95	66.18
	30	75.22	86.90	64.45	78.22
	*35*	*100.00*	*100.00*	*98.57*	*100.00*
	*40*	*99.34*	*99.55*	*100.00*	*99.90*

Skimmer voltage (V)	40	45.18	47.42	47.96	43.03
	50	86.51	60.99	93.69	81.45
	*60*	*99.38*	*100.00*	*95.25*	*97.76*
	*65*	*100.00*	*98.69*	*100.00*	*100.00*
	70	92.17	50.10	97.32	98.33

**Table 3:** Method validation parameters for the studied model alkaloids: linearity, precision, limits of detection, and quantification.

Compound	Regression equation	*R* ^2^	Linearity range (µg/mL)	LOD (ng/mL)	LOQ (ng/mL)	Intraday precision (*n* = 5) [%]	Interday precision (*n* = 5) [%]
Berberine	*y* = 363158032093*x* + 856955661	0.998	0.1–100	47	157	2.6	3.1
Berbamine	*y* = 55985070215*x* + 4453198619	0.997	0.1–250	25	84	3.4	4.2
Magnoflorine	*y* = 35358955125*x* + 9667117613	0.998	0.1–100	18	61	1.8	2.1
Sanguinarine	*y* = 7879756395*x* + 48208329	0.997	0.1–100	22	73	1.2	1.4

**Table 4:** The behaviour of model alkaloids in different collision energies together with the abundance of obtained signals.

Number	Alkaloid	Precursor ion	Major fragments obtained at various CID voltage (V) with abundances in different intensities (%)	
(1)	Berberine	336	10 V	336 (100), 337 (27), 321 (5)
			20 V	336 (100), 321 (43), 292 (22)
			30 V	**320 (100), 292 (73), 321 (69), 306 (26)**
			40 V	320 (100), 278 (55), 292 (49), 318 (31), 306 (25)
			50 V	278 (100), 318 (38), 292 (31), 279 (2)
			60 V	278 (100), 263 (31), 249 (27), 220 (26)
			80 V	191 (100), 220 (47), 204 (46), 192 (38)

(2)	Magnoflorine	342	10 V	342 (100), 343 (31), 297 (12), 58 (10)
			20 V	297 (100), 265 (85), 342 (78), 58 (71)
			30 V	**58 (100), 265 (29), 297 (16)**
			40 V	58 (100), 191 (31), 222 (29)
			50 V	58 (100), 179 (20), 192 (10)
			60 V	58 (100), 165 (56), 176 (20)
			80 V	58 (100), 165 (50), 178 (40)

(3)	Sanguinarine	332	10 V	332 (100), 333 (27), 334 (3)
			20 V	332 (100), 333 (24), 304 (7)
			30 V	**332 (100), 274 (41), 304 (39), 317 (34)**
			40 V	274 (100), 304 (60), 246 (52), 216 (30)
			50 V	304 (100), 216 (55), 246 (48), 189 (22)
			60 V	246 (100), 216 (60), 260 (40), 189 (30)
			80 V	189 (100), 202 (85), 176 (75)

(4)	Berbamine	609	10 V	609 (100), 610 (38), 611 (35), 612 (16)
			20 V	609 (100), 611 (76), 206 (29), 579 (12)
			30 V	**609 (100), 611 (80), 192 (80), 578 (69), 206 (64), 296 (45)**
			40 V	206 (100), 192 (93), 174 (36), 190 (34), 296 (34)
			50 V	206 (100), 192 (90), 296 (52), 239 (41)
			60 V	206 (100), 192 (62), 174 (47), 296 (40)
			80 V	160 (100), 191 (94), 165 (85), 174 (84)

Note: the most optimal parameters are given in bold.

**Table 5:** Quantitative analysis of some major constituents in the studied extracts.

Extract number	% BBR	SD	% JTR	SD	% PLT	SD	% STP	SD	% SNG	SD	% CHD	SD	% CHE	SD	% BBM	SD	% OBB	SD	% ARM	SD	% NMC	SD	% MGN	SD
1	1.038	0.09	NT	—	TR	—	NT	—	0.968	0.00	2.608	0.11	2.346	0.02	NT	—	NT	—	TR	—	NT	—	1.689	0.08
2	2.859	0.15	NT	—	NT	—	NT	—	TR	—	0.438	0.03	2.497	0.01	NT	—	NT	—	0.230	0.01	NT	—	NT	—
3	1.071	0.05	2.413	0.11	0.048	0.00	NT	—	0.313	0.02	0.767	0.04	0.165	0.01	NT	—	NT	—	TR	—	NT	—	NT	—
4	NT	—	3.061	0.31	3.100	0.11	NT	—	1.809	0.08	NT	—	0.170	0.01	NT	—	TR	—	2.394	0.13	NT	—	1.475	0.21
5	3.583	0.17	0.410	0.04	0.039	0.00	NT	—	0.089	0.01	NT	—	0.708	0.01	NT	—	TR	—	0.086	0.01	NT	—	NT	—
6	3.013	0.19	2.248	0.09	0.033	0.00	NT	—	0.175	0.01	NT	—	0.365	0.02	NT	—	NT	—	0.101	0.01	NT	—	1.983	0.11
7	1.312	0.04	NT	—	0.028	0.00	NT	—	0.195	0.02	NT	—	0.218	0.02	TR	—	0.112	0.01	0.566	0.03	NT	—	NT	—
8	2.700	0.29	NT	—	1.249	0.01	NT	—	0.150	0.01	TR	—	0.649	0.04	0.208	0.01	0.179	0.01	TR	—	NT	—	0.654	0.02
9	2.467	0.15	NT	—	0.087	0.00	NT	—	0.165	0.01	TR	—	0.584	0.02	NT	—	NT	—	0.001	0.00	1.530	0.06	1.621	0.13
10	1.520	0.06	NT	—	0.047	0.00	NT	—	0.047	0.01	NT	—	0.488	0.01	NT	—	NT	—	0.177	0.01	0.300	0.02	NT	—
11	1.985	0.07	1.420	0.07	0.249	0.00	NT	—	0.279	0.01	NT	—	0.199	0.02	NT	—	NT	—	3.042	0.09	0.077	0.01	0.473	0.03
12	1.361	0.11	4.616	0.35	0.022	0.00	3.764	0.04	0.167	0.01	TR	—	0.064	0.00	NT	—	NT	—	0.320	0.02	NT	—	NT	—
13	0.506	0.02	0.572	0.16	NT	—	1.254	0.08	0.068	0.00	NT	—	TR	—	NT	—	TR	—	0.775	0.07	TR	—	0.611	0.02
14	2.969	0.08	0.642	0.02	0.005	0.00	NT	—	0.068	0.00	NT	—	0.228	0.01	NT	—	NT	—	3.220	0.15	NT	—	1.206	0.06
15	1.309	0.02	NT	—	TR	—	TR	—	0.481	0.03	NT	—	0.279	0.01	NT	—	NT	—	2.849	0.14	NT	—	NT	—
16	3.444	0.33	1.830	0.09	1.298	0.04	0.714	0.05	NT	—	TR	0.00	NT	—	1.756	0.08	0.449	0.02	0.499	0.03	0.425	0.03	3.279	0.07
17	2.096	0.13	2.206	0.20	0.287	0.02	TR	—	0.517	0.01	NT	—	2.609	0.19	0.183	0.01	0.142	0.01	0.277	0.02	TR	—	1.912	0.06
18	3.525	0.24	1.710	0.17	1.195	0.01	0.234	0.02	NT	—	NT	—	NT	—	1.301	0.09	0.144	0.01	1.525	0.07	NT	—	2.465	0.10
19	2.983	0.15	NT	—	5.668	0.05	0.136	0.01	NT	—	NT	—	TR	—	NT	—	0.337	0.02	TR	—	NT	—	NT	—
20	3.657	0.11	1.606	0.03	TR	—	0.712	—	1.261	0.11	NT	—	3.625	0.35	NT	—	TR	—	0.198	0.01	TR	—	NT	—
21	3.619	0.21	NT	—	0.923	0.05	0.127	0.01	0.324	0.03	1.566	0.09	0.287	0.02	NT	—	NT	—	TR	—	TR	—	1.306	0.05
22	1.841	0.03	NT	—	NT	—	NT	—	2.114	0.14	3.276	0.25	3.797	0.41	NT	—	TR	—	NT	—	NT	—	2.370	0.10
23	3.225	0.17	NT	—	1.104	0.00	0.150	0.01	0.289	0.01	1.568	0.09	0.471	0.02	NT	—	NT	—	NT	—	NT	—	0.213	0.01
24	5.382	0.41	2.231	0.09	1.815	0.21	0.358	0.01	NT	—	NT	—	NT	—	0.105	0.01	0.097	0.01	0.128	0.02	0.037	0.00	7.078	0.84
25	5.709	0.65	2.733	0.23	1.955	0.14	0.368	0.03	NT	—	NT	—	NT	—	2.169	0.24	0.335	0.02	1.368	0.01	NT	—	2.060	0.13

NT, not traced; TR, small quantity traced; BBR, berberine; JTR, jatrorrhizine; PLT, palmatine; STP, stylopine; CHD, chelidonine; CHE, chelerythrine; BBM, berbamine; OBB, obaberine; ARM, armepavine; NMC, N-methylcoclaurine; MGN, magnoflorine.

## Abbreviations

ARM: Armepavine
BBM: Berbamine
BBR: Berberine
CHD: Chelidonine
CHE: Chelerythrine
CID: Collision-induced dissociation
ISQA: Isoquinoline alkaloids
JTR: Jatrorrhizine
MGN: Magnoflorine
MS: Mass spectrometry
NMC: N-Methylcoclaurine
OBB: Obaberine
PLT: Palmatine
STP: Stylopine
TIC: Total ion current.
